# Exchange of Coordinated Solvent During Crystallization of a Metal–Organic Framework Observed by In Situ High‐Energy X‐ray Diffraction

**DOI:** 10.1002/anie.201600896

**Published:** 2016-03-09

**Authors:** Yue Wu, Matthew I. Breeze, Guy J. Clarkson, Franck Millange, Dermot O'Hare, Richard I. Walton

**Affiliations:** ^1^ Department of Chemistry University of Warwick Coventry CV4 7AL UK); ^2^ Department of Chemistry University of Oxford Oxford OX1 3TA UK; ^3^ Département de Chimie Université de Versailles-St-Quentin-en-Yvelines 45 Avenue des États-Unis 78035 Versailles cedex France

**Keywords:** crystal growth, host–guest systems, metal–organic frameworks, microporous materials, X-ray diffraction

## Abstract

Using time‐resolved monochromatic high energy X‐ray diffraction, we present an in situ study of the solvothermal crystallisation of a new MOF [Yb_2_(BDC)_3_(DMF)_2_]⋅H_2_O (BDC=benzene‐1,4‐dicarboxylate and DMF=N,N‐dimethylformamide) under solvothermal conditions, from mixed water/DMF solvent. Analysis of high resolution powder patterns obtained reveals an evolution of lattice parameters and electron density during the crystallisation process and Rietveld analysis shows that this is due to a gradual topochemical replacement of coordinated solvent molecules. The water initially coordinated to Yb^3+^ is replaced by DMF as the reaction progresses.

The synthesis of metal–organic frameworks (MOFs) has, to date, been a process fraught with assumptions, due to the difficulty of obtaining high quality structural data in situ during their formation that would provide detailed information about their crystallisation mechanism.^1^ Although studies of kinetic vs. thermodynamic control in the synthesis of MOFs have been reported by screening products of reactions isolated as a function of time, using both experimental and theoretical approaches,^2^ an understanding of the early stages of MOF crystallisation processes remains poor. One untested assumption is that after a MOF nucleates, it crystallises without undergoing further structural changes. The functionality of many metal‐organic framework materials derives from their ability to interact with guest molecules. In MOFs in which the metal coordination sphere is not fully saturated with structural ligands, the interaction between metal and coordinated molecules tends to be particularly strong, and this may give rise to favorable adsorption and catalysis properties.^3^ Any interaction between guest and material must necessarily result in some level of change to the observed electron density distribution and unit cell size. This effect is prominent in several of the most widely studied MOFs: for example, the dehydroxylated UiO‐66 framework loses hydroxyl and it unit cell contracts by ca. 0.05 Å,^4^ while the difference between the guest‐bound and bare MOF‐74/CPO‐27 frameworks is on the order of 0.1 Å for both axes of the hexagonal cell.^5^ These changes are well within the range that can be clearly resolved using high‐resolution powder diffraction, and indeed this method has been used extensively in structural studies of the effect of adsorbed molecules on MOFs under gas atmospheres.^6^

In many cases of MOF synthesis using solvothermal methods, it is unclear whether the framework is initially formed with coordinated solvent that is then exchanged with another ligand to reach the final product, or if the final product is formed from the start as the only species. This knowledge would be valuable to the large scale deployment of MOFs, allowing the optimization of syntheses to reduce or eliminate the need for certain types of post‐synthetic processing, such as the high‐temperature dehydroxylation of UiO‐66.

Energy‐dispersive X‐ray diffraction (EDXRD) has been used to great effect to follow solvothermal crystallization of MOFs,^7^ building on earlier work on hydrothermal zeolite and zeotype formation.^8^ Here, using X‐rays without monochromation provides sufficient intensity to observe crystallisation in large‐volume reaction vessels, but with the serious disadvantage of the intrinsic low resolution of energy‐discriminating solid‐state detectors. Thus, although the changing intensity of well‐resolved Bragg peaks can be monitored in real time to yield crystallisation curves, it is difficult to observe and quantify small changes in unit cell parameters and impossible to perform atomistic (i.e. Rietveld) refinement, severely limiting the level of structural information available. More recently, advances in technology have made monochromatic XRD feasible. Recent work has used in situ monochromatic diffraction to study the mechanochemical^9^ and solvothermal^10^ synthesis of MOFs and while it has been shown that scale factors (phase fractions) and peak positions can be extracted, no full structural treatment has yet been performed of the temporal data measured in situ. Another great challenge in in situ studies is the trade‐off between reactor size and data quality: a larger reactor will provide conditions comparable to conventional, laboratory‐scale chemistry, while a capillary will provide optimal data quality but is severely limiting in terms of reproducing realistic synthetic conditions.

In this work, we analyse a MOF crystallization taking place within a stirred reaction tube of relatively large volume (ca. 5 mL, 9 mm diameter) using high intensity monochromatic radiation. We are able to obtain high‐quality data in situ under reaction conditions similar to those used in a conventional large‐scale batch synthesis. Not only do we obtain detailed kinetic information with exceptional time resolution, we are also able to observe the exchange of labile coordinated solvent within a framework material *during its formation*, which we can quantify using Rietveld analysis of the data measured in situ; this allows refinement of crystal structure as the reaction proceeds. Our results demonstrate a significant advance in the quality of diffraction data from crystallising material, obtained under solvothermal conditions from a relatively large‐scale synthesis.

The material investigated herein is a new MOF [Yb_2_(BDC)_3_(DMF)_2_]⋅H_2_O (BDC=benzene‐1,4‐dicarboxylate and DMF=*N*,*N*‐dimethylformamide) prepared under solvothermal conditions, from mixed water/ DMF solvent. The structure was solved and refined using single‐crystal analysis (see the Supporting Information (SI)). The framework crystallizes in the monoclinic *C*2/*c* space group and contains chains of Yb and carboxylate, related to a previously reported Er‐BDC framework.^11^ The chains run down the *c*‐axis of the framework, and are located at the corners of diamond‐shaped 1D channels. Running along each channel are the labile coordination sites of the Yb, which are occupied by DMF in the equilibrium structure, Figure 1. The material can be thermally desolvated to yield a permanently porous framework: a full characterisation is provided in the SI.

**Figure 1:**
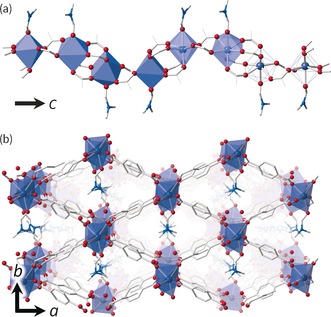
Structural representations of the material studied in fully DMF‐exchanged form [Yb_2_(BDC)_3_(DMF)_2_]⋅H_2_O. a) Undulating 1D metal‐carboxylate chains run down the *c* axis, with coordinated DMF hanging into the channels. b) Viewed down the *c* axis, the diamond shaped channels can be clearly seen. Yb atoms are purple, oxygen red, nitrogen blue and carbon black. For clarity hydrogen atoms are not shown, broken off bonds represent bridging BDC, and only the major orientation of DMF is shown.

In situ X‐ray diffraction data during the reaction of Yb chloride hydrate and BDC in mixed water/ DMF were collected at three temperatures (90, 110 and 120 °C), with patterns being collected at 30 s intervals. Unless otherwise stated, the data presented in the main text are from the 120 °C reaction; the other data follow similar trends, and are included in the SI. Using sequential Pawley refinements of each pattern, we simultaneously extract both lattice parameters, and total quantity of crystalline material through the integrated area beneath peaks (Figure 2). Figure 3 shows the concurrent changes seen in crystalline quantity and cell parameters. The crystalline quantity can be analysed to extract kinetic information (see SI), but the temporal shift in lattice parameters reveals the evolution of structure during crystallisation.

**Figure 2:**
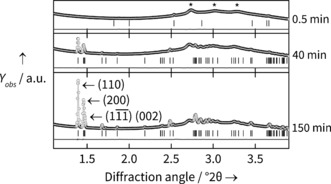
Data from the Yb‐BDC synthesis at 120 °C, showing examples of Pawley fits for no diffraction (0.5 min), low diffraction (40 min) and strong diffraction (150 min) cases. Residuals and tick marks for H_2_BDC (0.5 min) and Yb‐BDC (40, 150 min) are shown below the main plots. *λ*=0.2242 Å (55.3 keV).

**Figure 3:**
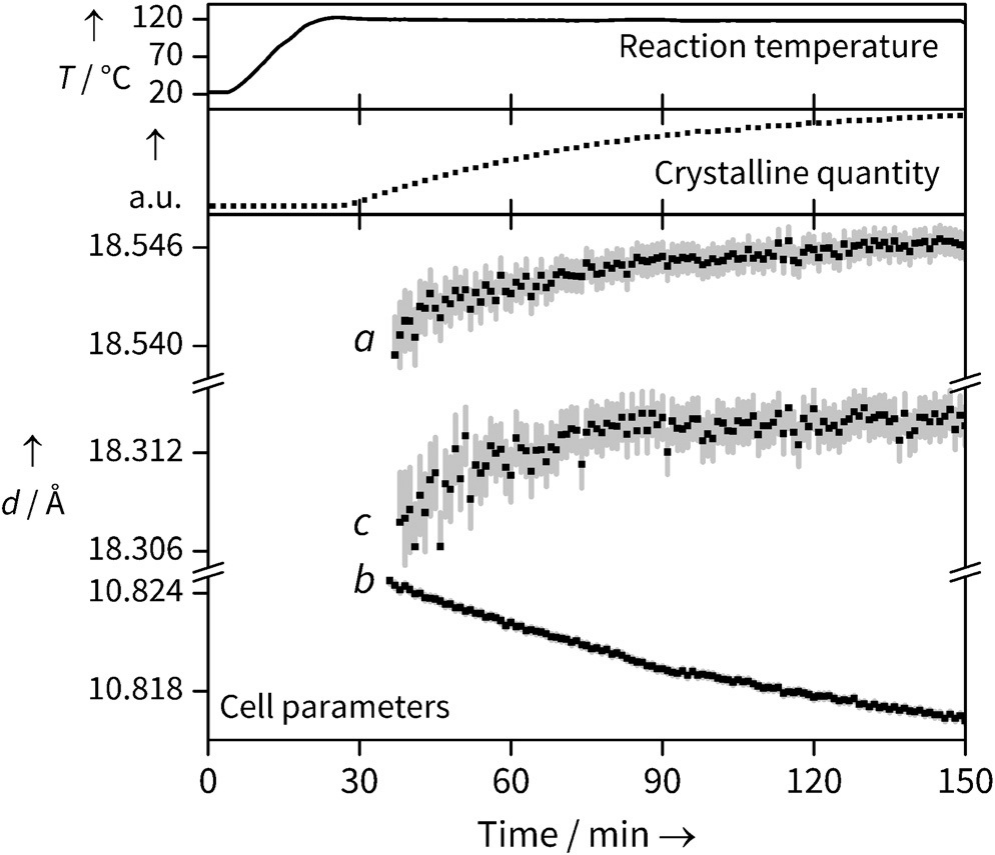
Data from the Yb‐BDC synthesis at 120 °C, showing the simultaneous extraction of changes in total crystalline quantity (normalized units obtained from full pattern integration) and unit cell parameters (error bars are shown to 1 e.s.d.). Temperature readings from an internal thermocouple show that the growth occurs under isothermal conditions.

The changes in lattice parameter are small, but changes on the order of 0.001 Å are easily and reproducibly resolved, and show a meaningful trend with temperature (see SI). It should be noted that the internal thermocouple shows that temperature is reached prior to the observation of Bragg peaks (Figure 3) so thermal effects on lattice parameters can be ruled out. In fact, their evolution continues with the same trend throughout the crystallisation, so we are confident the crystalline material is seen under isothermal conditions. We also note the Bragg peak widths do not decrease significantly during the period of analysis so we rule out changing crystallite size as a significant effect on lattice parameter evolution.

As the reaction progresses, an increase in the unit cell lengths can be seen for the *a* and *c* cell parameters, while the *b* parameter decreases; this is shown in Figure 3. As the *a* and *b* cell parameters correspond to the diagonals of the diamond shaped channels, expansion in one direction must be countered by contraction in the other. Such behaviour is expected for a diamond shape in which the interior angles can change freely but the side lengths are constrained, and is reminiscent to that seen in the “breathing” MOF MIL‐53, which also has diamond‐shaped one‐dimensional channels where the introduction of weakly bound molecules, or application of temperature or pressure, causes similar changes in the relative pore dimensions,^12^ although the evolution of lattice parameters for our material is several orders of magnitude smaller.

A sequential Rietveld refinement was performed on the individual patterns. The occupancy of all atoms in the DMF moiety was linked to a single parameter and allowed to freely refine, except for the oxygen atom which was fixed at occupancy 1, as this site is occupied by oxygen regardless of H_2_O or DMF coordination. The results agree very well with the temporal evolution of the ratio of the areas of the (200) and (110) peaks obtained from Pawley refinement, shown in Figure 4 a for the 120 °C data set. This is consistent with the fact that the DMF electron density lies primarily on the crystallographic (200) plane; Figure 4 b shows how the powder patterns are sensitive to the nature of coordinated solvent. The magnitude of change of DMF occupancy during crystallisation is not as great as that going from the solely Yb‐OH_2_ to solely Yb‐DMF, but the simulation does not take into account non‐coordinated solvent, which may still contribute to the electron density. During the period of the in situ analysis it more likely that the solvent simply is changing from water‐rich to DMF rich rather than representing complete exchange of one by another.

**Figure 4:**
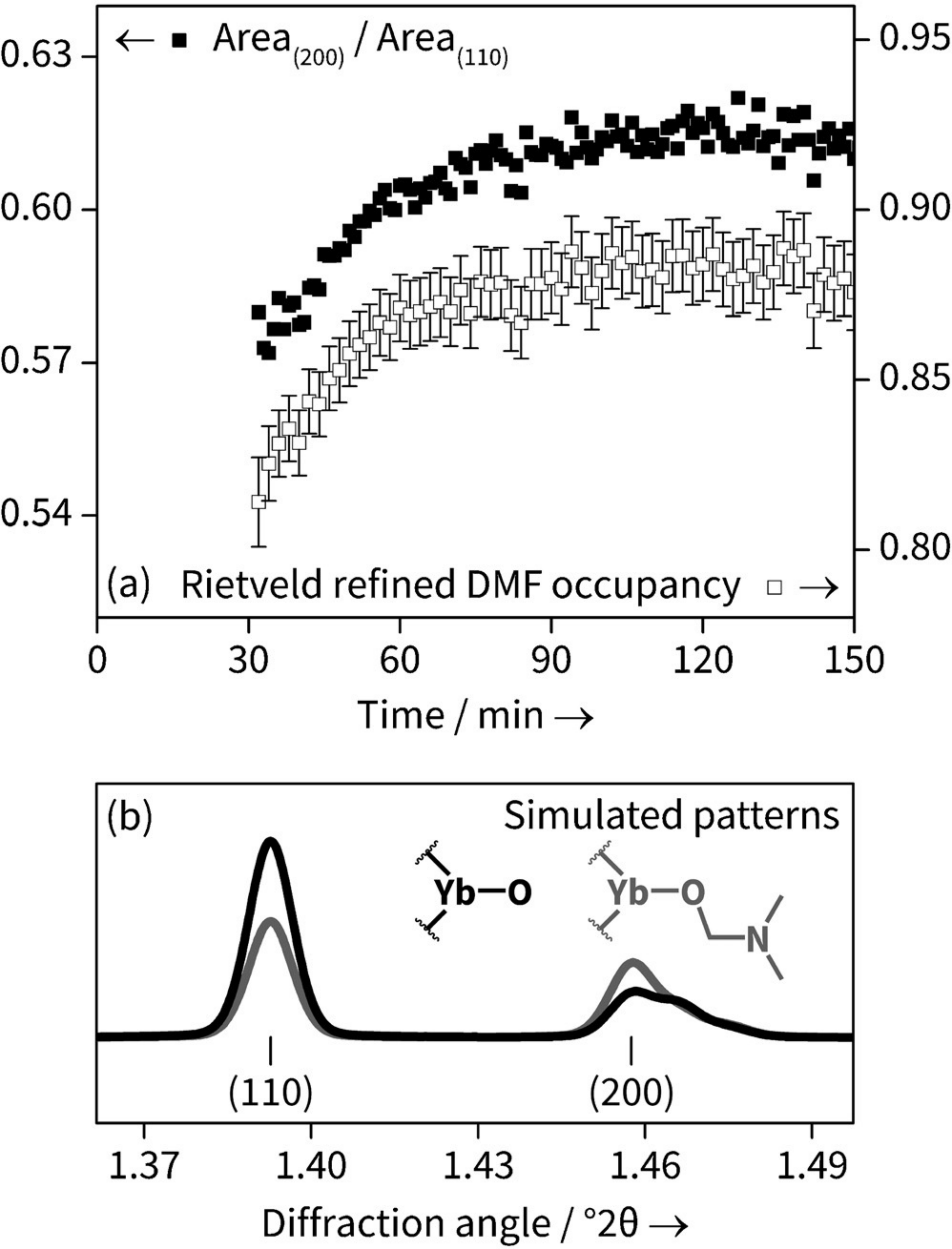
a) Plot of the ratio of the areas of (200) and (110) peaks obtained from Pawley refinement with the refined occupancy of a nitrogen atom representing amount of coordinated DMF (for clarity, only every second data point is shown for each data set) and b) simulated powder patterns showing the effect of solvent exchange showing the cases for 100 % water occupancy and 100 % DMF occupancy.

To confirm the reason for the changing structural parameters of the Yb‐BDC material during its formation, combined thermogravimetric analysis, differential scanning calorimetry and mass spectrometry (TGA‐DSC‐MS) experiments were performed on quenched samples prepared in the same sized, stirred reaction vessel used in the in situ studies but heated in an oil bath at 120 °C for three durations (30, 45 and 60 mins) within the timescale of crystallisation seen in the in situ experiments. This showed distinct differences in the solvent loss steps (Figure 5 and SI). The TGA trace of the 30 minutes sample shows considerably more surface water (no DMF is lost at this stage, as shown by the MS data) and the subsequent bound solvent loss is less well defined, perhaps suggesting water is lost from the bulk as well as the surface. More significantly, the DSC traces for the pair of events between 140 and 260 °C, which correspond to bound solvent loss, show small shifts to higher temperatures as the sample synthesis time is increased. This would be consistent with a different solvent composition in the solids as synthesis time is increased. The most striking evidence for a changing solvent composition, however, comes from the MS traces: as seen in Figure 5 c the relative amount of DMF lost in each of the two solvent loss features shows a systematic change in ratio, entirely consistent with less directly bound DMF being present in the samples quenched at shorter reactions times. With the caveat that quenching studies will always carry the risk that the material recovered undergoes some irreversible change upon cooling and extraction from the solvent, such as exchange of water with the air, our TGA‐DSC‐MS results provide important corroborative evidence for the conclusions from the in situ study.

**Figure 5:**
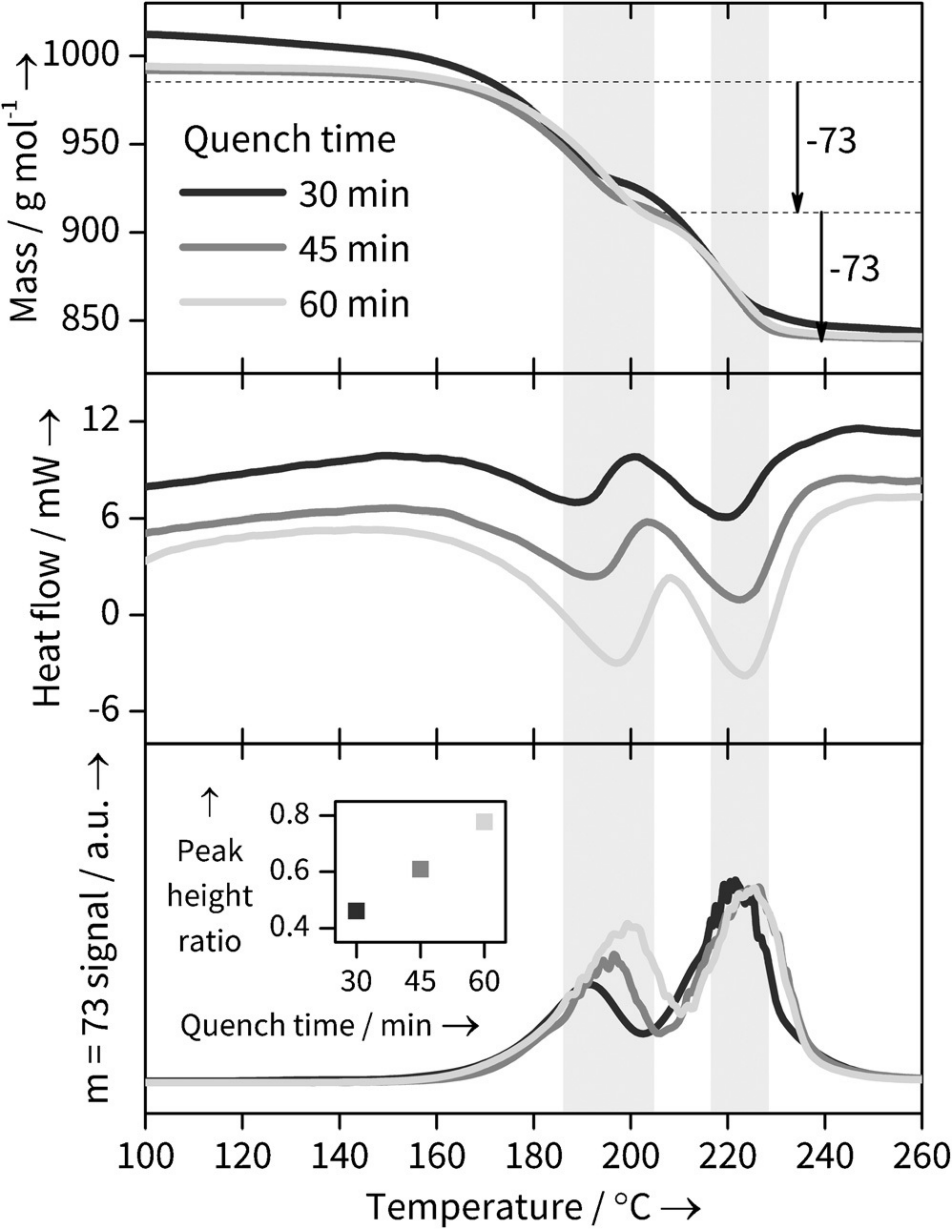
TGA‐DSC‐MS evidence for different solvation states in Yb‐BDC samples quenched after 30, 45 and 60 min. Shaded regions highlight the expected loss of 2 bound DMF molecules. a) TGA data normalised against fully desolvated Yb‐BDC mass showing expected DMF (*m*=73) loss steps. b) DSC data showing change in the DSC troughs corresponding to solvent loss. c) MS *m*=73 signal corresponding to DMF loss and (inset) ratio of two peak heights, showing changing DMF solvation with reaction time.

Thus we construct a consistent model for the evolution of lattice parameters in which solvent exchange takes place *during* the formation of the material. At the early stages of reaction the material is water rich, and the directly coordinated water is replaced by DMF as the reaction proceeds, with the framework geometry adjusting to account for the change in the size and shape of the occluded molecules. Thus the chemical composition [Yb_2_(BDC)_3_(solvent)_2_]⋅solvent (where solvent=H_2_O and/or DMF) is a general representation of the materials formed in the solvothermal reactions, with the ultimate product being [Yb_2_(BDC)_3_(DMF)_2_]⋅H_2_O, the single crystal studied that was prepared using a considerably longer reaction time than the in situ experiments.

We have demonstrated that, under conditions close to those used for conventional MOF synthesis, it is possible to observe solvent exchange in situ during synthesis and to extract quantitative information regarding composition from Rietveld analysis. While capillaries have previously been effectively used by others to study the solvothermal crystallisation of inorganic materials in situ,^13^ our use of a large volume reactor (5 mL) has the distinct advantage of allowing reagents to be easily added in homogeneous, pre‐planned quantities to ensure reproducibility; this is particularly important when solid/liquid mixtures are investigated that are difficult to transfer into capillaries in desired quantities. Our observation of solvent exchange *during* crystallisation suggests a previously unexplored method of optimizing synthetic parameters for control of composition of MOFs in both large‐scale MOF deployment and lab‐scale reactions. Our in situ XRD approach would also be valuable in the study of other MOF formation processes, for example, in determining the rate at which different metals or ligands incorporate into a solid‐solution (“multivariate”) MOF: the difference in cell parameters between isostructural phases is well above the smallest changes that can be observed. For example, in the work of Lin Foo et al., there is around 0.1 Å difference between the end members of a mixed‐ligand series, and the cell parameter change closely follows Vegard's law;^14^ in the work of Yeung et al., who studed a three‐ligand solid solution, this difference is close to 0.5 Å.^15^

## Experimental Section

[Yb_2_(BDC)_3_(DMF)_2_]⋅H_2_O was synthesised under solvothermal conditions (BDC=benzene‐1,4‐dicarboxylate and DMF=*N*,*N*‐dimethylformamide). Ytterbium(III) chloride hexahydrate (1 mmol) and benzene‐1,4‐dicarboxylic acid (15 mmol) were dissolved in DMF (5 mL). To this, H_2_O (0.15 mL) was added and the mixture stirred until complete dissolution of all reagents had occurred. The reactants were heated in a sealed 20 mL Teflon‐lined autoclave at 100 °C for 20 hours. The resulting white crystalline solid was isolated by suction filtration. In situ crystallisation studies were carried out on Beamline I12 (JEEP) of the Diamond Light Source.^16^ A specially constructed reaction cell made from polyether ether ketone (PEEK) was used to investigate solvothermal crystallisation: a 5 mL internal volume tube of 12 mm internal diameter that was fitted with a screw‐top lid that allowed moderate pressure to be contained and reactions up to 150 °C to be investigated. An internal thermocouple, threaded through the lid of the reaction tube allowed continuous monitoring of temperature during reactions. The reaction was stirred rapidly with a smaller Teflon‐coated magnetic follower to aid heat transfer and to ensure that uniform solid product was present in the X‐ray beam throughout the experiment. The tube was heated within the ODISC infra‐red furnace,^17^ with a glassy carbon sheath around the sample to allow heat transfer to the reaction vessel. A wavelength of 0.2242 Å was used and 2D diffraction patterns collected every minute using a Pixium image plate detector (430×430 mm^2^) with an exposure time of 4000 ms. The system was calibrated with a crystalline CeO_2_ reference and the 2D image plate data were integrated using the fit2d software to give 1D diffraction patterns.^18^ The time‐resolved in situ data sets were analysed using sequential Pawley decompositions and Rietveld refinements, as implemented in TOPAS.^19^ CCDC 1057461 contain the supplementary crystallographic data for this paper. These data can be obtained free of charge from The Cambridge Crystallographic Data Centre.

## Supporting information

As a service to our authors and readers, this journal provides supporting information supplied by the authors. Such materials are peer reviewed and may be re‐organized for online delivery, but are not copy‐edited or typeset. Technical support issues arising from supporting information (other than missing files) should be addressed to the authors.

Supplementary
