# Supplementary material for: Exchange of Coordinated Solvent During Crystallization of a Metal–Organic Framework Observed by In Situ High‐Energy X‐ray Diffraction
Source: Angew Chem Int Ed Engl. 2016 Mar 9;55(16):4992–6. doi: 10.1002/anie.201600896 (PMC6680260; doi:10.1002/anie.201600896)
Supplement: Supplementary file 1 — Supplementary [file ANIE-55-4992-s001.pdf]

Angewandte Chemie
Eine Zeitschrift der Gesellschaft Deutscher Chemiker

Supporting Information

Exchange of Coordinated Solvent During Crystallisation of a Metal–Organic Framework Observed by In Situ High Energy X-ray Diffraction

*Yue Wu[+], Matthew I. Breeze[+], Guy J. Clarkson, Franck Millange, Dermot O'Hare, and Richard I. Walton**

anie_201600896_sm_miscellaneous_information.pdf

SUPPORTING INFORMATION

Contents

S1: Single Crystal Data Analysis of [$Yb_2(BDC)_3(DMF)_2$]·H_2O 2

S2: *In situ* Powder XRD – Pawley Analysis 3

S3: TGA-DSC-MS data 6

S4: Kinetic Analysis 11

S5: Study of the Desolvated Phase $Yb_2(BDC)_3$ 16

S6: BET Analysis of nitrogen adsorption from $Yb_2(BDC)_3$ 21

S7: *In Situ* Powder XRD – Rietveld analysis 22

S8: References 25

S1: Single Crystal Data Analysis of $[Yb_2(BDC)_3(DMF)_2]\cdot H_2O$

A needle crystal was selected and cut into a block. An Oxford Diffraction Gemini four-circle system with Ruby CCD area detector with (Mo Kα) radiation was used for data collection with the crystal held at 100(2) K with an Oxford Cryostream Cobra. Systematic absences indicated space group *Cc* or *C*2/*c* and the latter was chosen on the basis of intensity statistics and shown to be correct by successful refinement. The crystal chosen had a twin component. Matrices relating the two twin components were:

Twin 1 1.0000 0.0000 0.0000 0.0000 1.0000 0.0000 0.0000 0.0000 1.0000

Twin 2 -0.9994 -0.0001 -0.4142 0.0001 -1.0001 0.0000 -0.0001 0.0001 0.9994

The two orientations are related by a rotation = -179.9892 deg around the following vectors:

Reciprocal space (hkl): -0.2036 0.0002 0.9790

Direct space (uvw) : -0.0001 -0.0000 1.0000

In the twinned data refinement the scale factors (twin ratios) were 0.8746(5) : 0.1254(5). The integration of the data was conducted with the twinning suite in the Crysalis Pro software and refined with Olex2. The structure was solved by direct methods using SHELXS with additional light atoms found by Fourier methods. Hydrogen atoms were added at calculated positions and refined using a riding model with freely rotating methyl groups. Anisotropic displacement parameters were used for all non-H atoms; H atoms were given isotropic displacement parameters equal to 1.2 (or 1.5 for methyl hydrogen atoms) times the equivalent isotropic displacement parameter of the atom to which the H-atom is attached. The asymmetric unit contains a Yb, a complete 1,4-benzenedicarboxylate, a 1,4-benzenedicarboxylate lying on a twofold axis through its short axes and a coordinated DMF. Additionally there is some electron density modelled as a partially occupied water molecule (see below). The coordinated DMF model had long cigar shaped thermal ellipsoids so was modelled as disordered over two positions. The occupancy of the two orientations was allowed to refine freely but was then fixed at 60:40 for the final stages of refinement. A restraint was used to give the minor component similar bond lengths and angles to the main component DMF. Some electron density located in a void was modelled as a water molecule at 50% occupancy. No hydrogens were located on this water but were included in the final formula so as to calculate the correct density.

Table S1.1: Crystal data for $[Yb_2(BDC)_3(DMF)_2]\cdot H_2O$

Data Collection	Oxford Diffraction Gemini (Mo Kα)
Temperature / K	100(2)
Crystal System	monoclinic
Space Group	*C*2/*c*
a / Å	18.2950(4)
b / Å	10.8482(2)
c / Å	17.3984(4)
α / °	90
β/ °	101.395(2)
γ/ °	90
V / Å^3	3384.96(12)
Z	4
μ(Mo Kα) / mm^{-1}	5.565
Crystal size / mm^3	0.18 x 0.16 x 0.14
R_{int}	0.0267
Reflections measured, unique	8841, 8841
GOF on F^2	1.030
$R1$, wR_2 [$I > 2\sigma(I)$]	0.0358, 0.1042
Data / restraints / parameters	8841/32/232
Largest peak, hole / e·Å^{-3}	2.64/-1.46

S2: *In situ* Powder XRD – Pawley Analysis

Full pattern analysis of powder patterns was performed using the Pawley method within the TOPAS[1] software to determine lattice parameters.

Selected refinements were also inspected manually to confirm the validity of the sequential method. The background was modelled using a freely refining eight-term Chebyshev polynomial. The integrated area of background-subtracted Bragg peaks (obtained through the TOPAS “crystalline_area” function) was used to provide the measure of total sample crystallinity. Broad peaks from the PEEK reaction vessel, Figure S2.1, were modelled by three individual peak shapes fitted to diffraction patterns in which no crystalline material was present. The positions and profile parameters of these peaks were then fixed, with intensity freely refining.

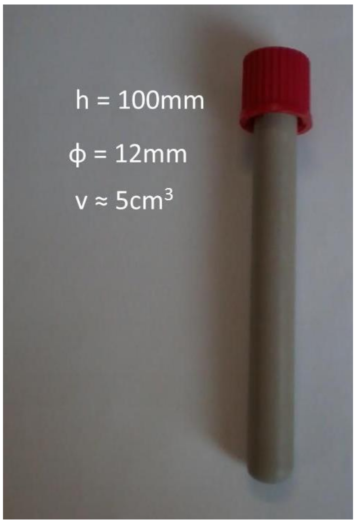

Figure S2.1: Photograph of PEEK reaction tube (thermocouple not shown)

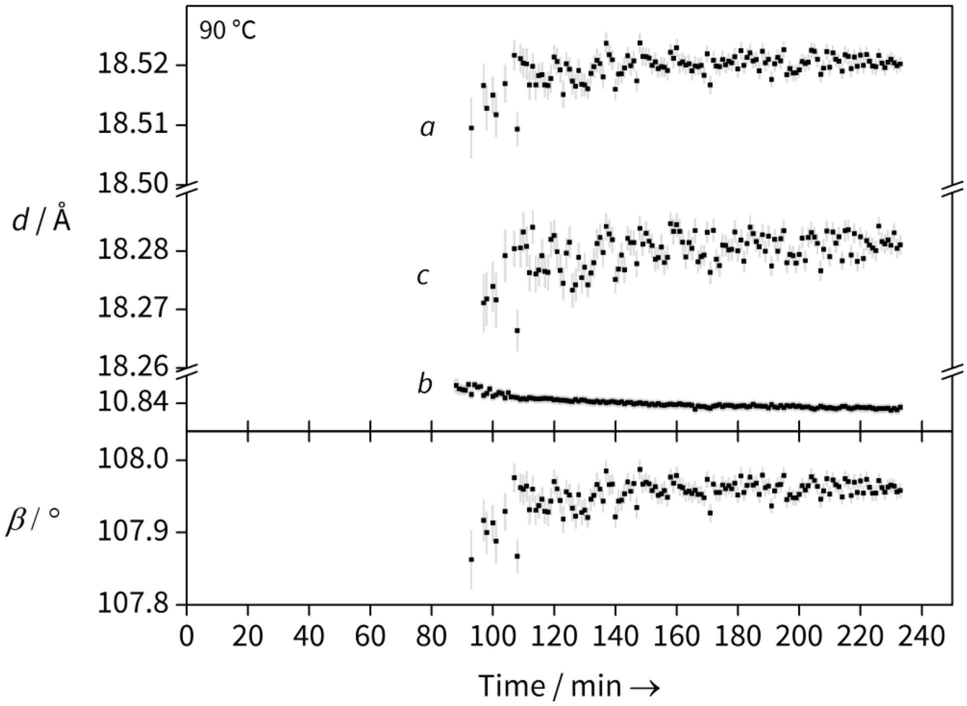

Figure S2.2: Cell parameters refined from *in situ* data at 90 °C; errors show 1 e.s.d.

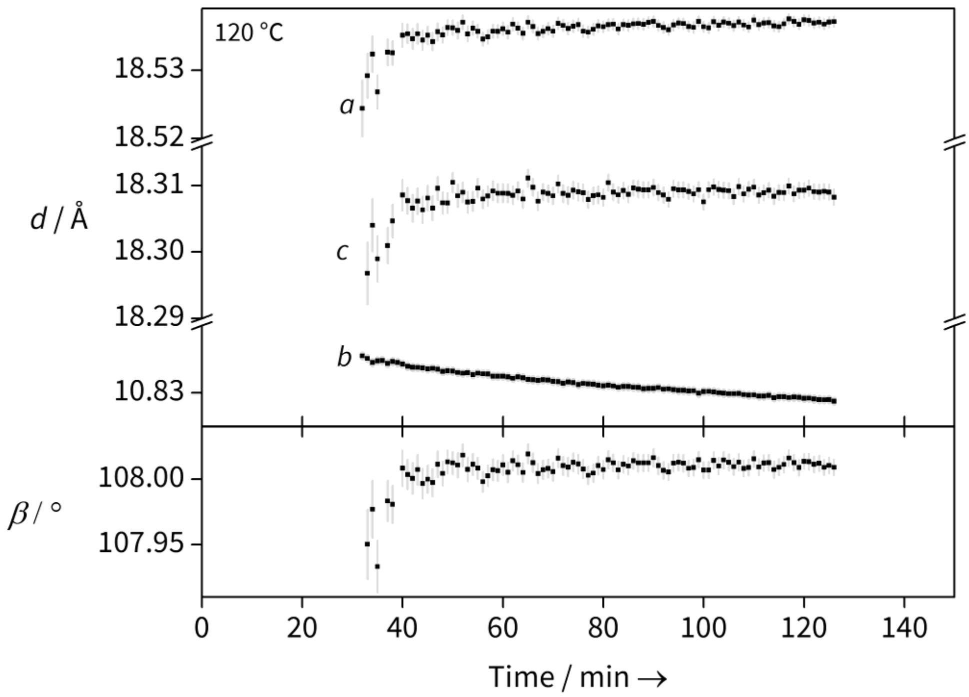

Figure S2.3: Cell parameters refined from *in situ* data at 110 °C; errors show 1 e.s.d.

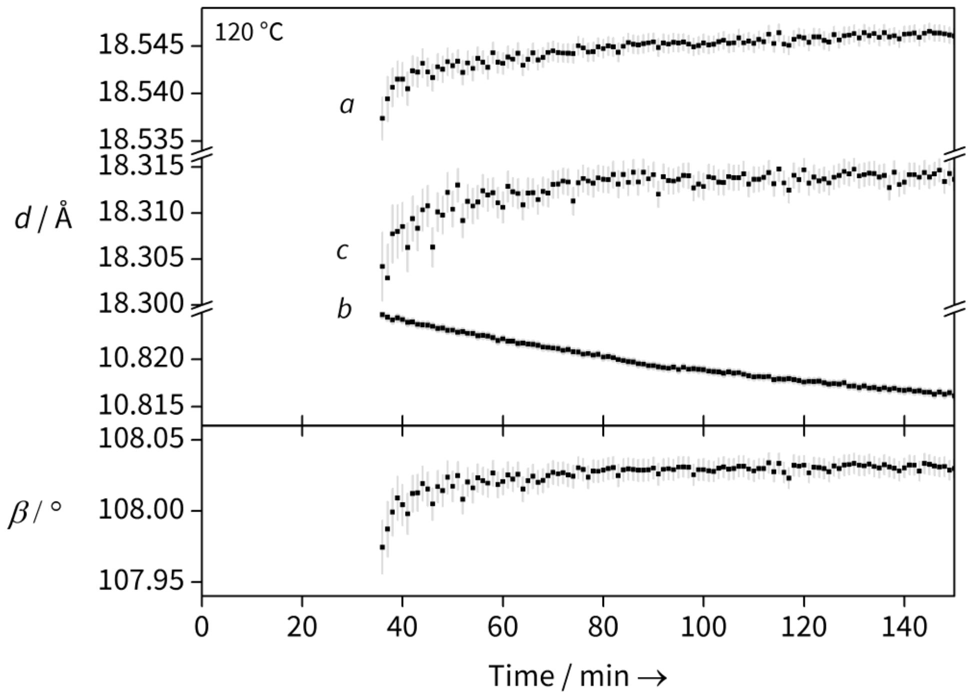

Figure S2.4: Cell parameters refined from *in situ* data at 120 °C; errors show 1 e.s.d.

S3: TGA-DSC-MS data

Simultaneous thermogravimetric analysis (TGA), differential scanning calorimetry (DSC) and mass spectrometry (MS) was used to study quenched samples. In order to mimic the conditions used for the *in situ* XRD experiments, reactions were performed in the same sized reaction tubes with stirring but in this case they were heated in an oil bath preheated to 120 ^{o}C. The reaction tubes were removed from heating at chosen times (30 minutes, 45 minutes and 60 minutes) and recovered immediately by suction filtration, while still hot (to avoid any precipitation of unreacted terephthalic acid) and allowed to dry on a filter paper in air for 3 minutes at room temperature before being transferred to sealed vials. The measurements were performed using a Mettler Toledo TGA/DSC 1-600 instrument with a Hiden HPR-20 QIC R&D specialist gas analysis system, a triple filter mass spectrometer with SEM detection on heating in nitrogen to 1000 °C at 10 °C min^{-1}. Nitrogen was chosen as carrier gas to minimise background water and to remove CO_2, and the traces to 400 °C were the same as those measured in air, the only difference being some incomplete combustion of the organic ligand to 1000 °C, as shown in Figures S3.1 and S3.2.

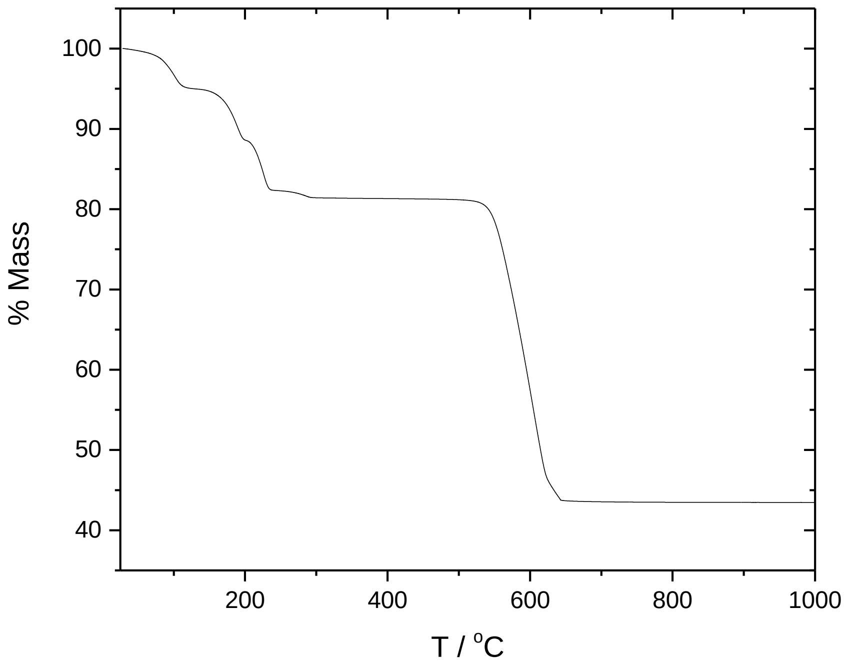

Figure S3.1: TGA trace of $Yb_2(DMF)_2(BDC)_3.H_2O$ measured in air (10 °C min^{-1})

Table S2.1: Analysis of TGA data of $Yb_2(DMF)_2(BDC)_3.H_2O$ produced after a 5 hour reaction.

	Temperature	Total % Mass Measured	Total % Mass Expected
$[Yb_2(DMF)_2(BDC)_3.H_2O].1.8H_2O$*	25 °C	100.00	100.00
$Yb_2(DMF)_2(BDC)_3$	135 °C	94.95	95.03
$Yb_2(DMF)(BDC)_3$	202 °C	88.50	87.90
$Yb_2(BDC)_3$	390 °C	81.32	80.76
Yb_2O_3	1000 °C	43.47	38.50

*the excess surface solvent could also be DMF, but TGA-DSC-MS results from the quenched samples show no DMF at low temperature: see Figure S3.3.

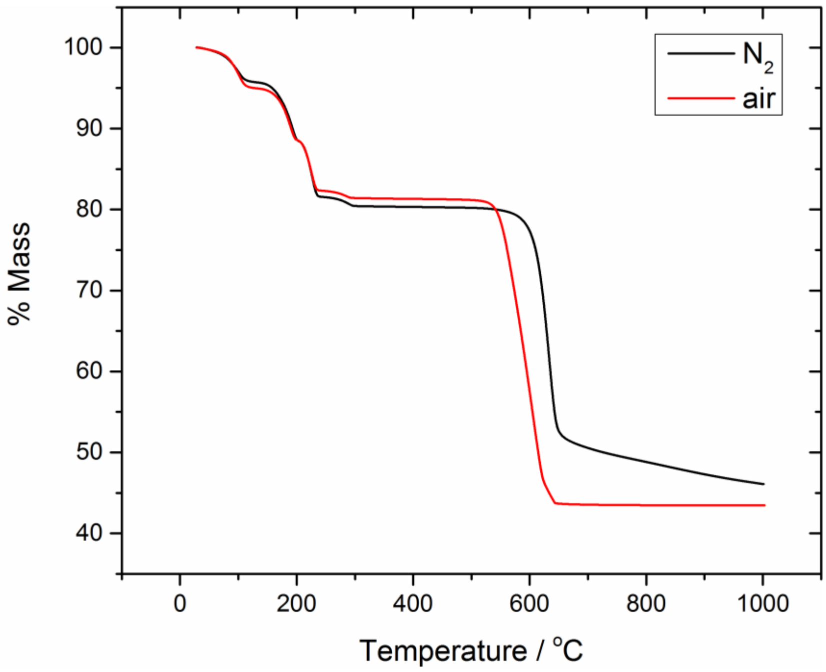

Figure S3.2: comparison of TGA measured in air and in nitrogen atmospheres for material prepared after 5 hours.

As the mass of the $Yb_2(BDC)_3$ framework provides a stable plateau in the TGA data, (remaining constant from ~200 to 600 °C), the TGA data were normalised against this value and plotted as an effective molecular weight in order to plot the expected mass losses arising from the loss of the two strongly bound ligands per formula unit, as shown in Figure S3.3. The change in mass loss arising from the varying solvation states

depending on reaction time are expected to be subtle – based on the Rietveld refinement, we expect to see an occupancy change of < 10 %, resulting in a relatively small mass change.

The MS is complicated by the fact that since DMF (mass 73) fragments to give, *inter alia*, masses 18 and 44.[2] In an attempt to distinguish H_2O and DMF, we collected MS data for m = 17, 18, 44 and 73. However, the data from these fragments were too weak to provide useable data. The m = 73 data were smoothed using the LOESS algorithm implemented in OriginPro 2015 (Figure S3.4).

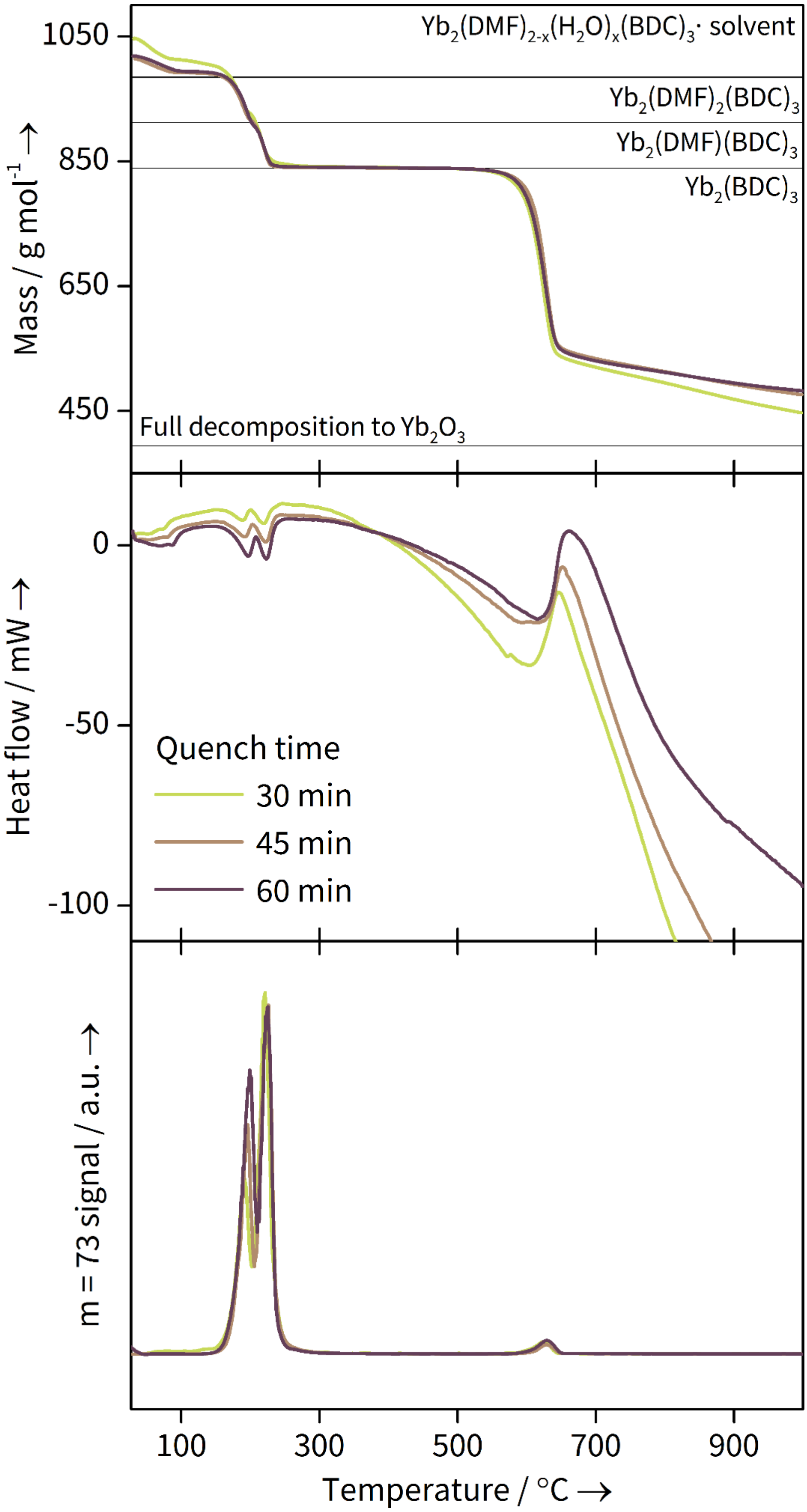

Figure S3.3: TGA-DSC-MS data of quenched Yb-BDC samples measured in N_2 (10 °C min^{-1})

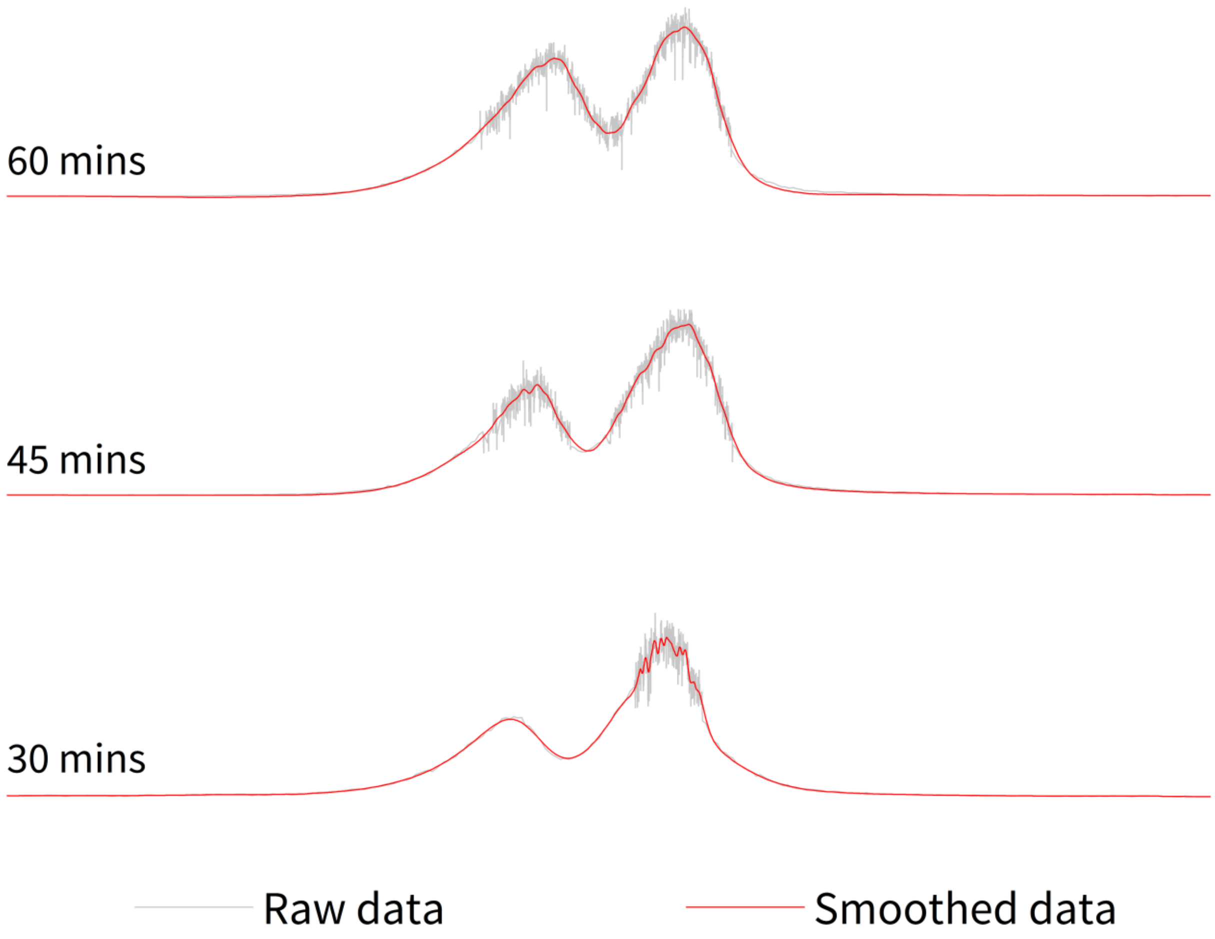

Figure S3.4: Raw and smoothed MS m = 73 data

S4: Kinetic Analysis

In situ XRD during solvothermal crystallisation was recorded at 3 temperatures: 90, 110 and 120 °C. Figure S4.1 shows 3D contour maps of the temporal evolution of Bragg scattering, along with the internal temperature of the reaction vessel as measured using the internal thermocouple. The plots show that at the early stages of reaction, Bragg peaks due to undissolved 1,4-benzenedicarboxylic acid are present, but these rapidly disappear before the $[Yb_2(BDC)_3(DMF)_2]\cdot H_2O$ product emerges smoothly. The reaction temperature is always reached before the appearance of $[Yb_2(BDC)_3(DMF)_2]\cdot H_2O$, and the heating profile for each temperature is very similar, which explains why the disappearance of 1,4-benzenedicarboxylic acid occurred at approximately the same time for each reaction studied. Full pattern fitting of the diffraction profiles within the TOPAS software (Figure 2, main article) allows extraction of the total Bragg peak area from the Pawley fit to give a direct measure of extent of crystallisation. Note that a smaller angular range was used to determine the total Bragg peak area to avoid the broad diffraction feature of the polymer tube giving inaccuracies in the values obtained. The crystallisation curves thus obtained were analysed using the kinetic model proposed by Gualtieri,[2] which has successfully been used to simulate nucleation-growth kinetics of a number of MOFs under solvothermal crystallisation.[3] In this model, nucleation is treated as a separate event to crystal growth so that two rate constants can be extracted, and the dimensionality of crystal growth is also a variable.

The Gualtieri equation may be written as:

$$\alpha = \frac{1}{1+\exp\left\{-\left(\frac{t-a}{b}\right)\right\}}.\left\{1-\exp\left[-\left(k_g t\right)^N\right]\right\} \qquad (1)$$

N is the dimensionality of crystal growth. k_g is the rate constant of crystal growth, while a and b are constants related to nucleation. Here there is no need to account for induction time as this is accounted for in the constants a and b.

Since not all of the reactions we studied reached completion, we included a normalisation factor, A, in our fitting to obtain the final fits shown in the paper. Thus we used the equation:

$$I = A\left[\frac{1}{1+\exp\left\{-\left(\frac{t-a}{b}\right)\right\}}.\left\{1-\exp\left[-\left(k_g t\right)^N\right]\right\}\right] \qquad (2)$$

Where *I* is the total Bragg peak intensity. Fitting was performed with least-squares refinements using the Origin software.

The rate constant for nucleation, k_n, is given by:

$$k_n = 1/a \qquad (3)$$

The probability of nucleation, P_N *vs* time can be calculated.

$$P_N = \exp\left\{-\frac{(t-a)^2}{2b^2}\right\} \qquad (4)$$

The relative magnitudes of k_g and k_n can then be used to infer which is the rate-determining process in crystallisation. In addition, the value of *b* can be used to deduce information about the nature of nucleation: if *b* ~ 15 nucleation is heterogeneous, if *b* ~ 20 nucleation is homogeneous, and if *b* > 20 then nucleation is autocatalytic.

Figure S4.2 shows the obtained fits to the crystallisation curves and Table S4.1 contains the fitted kinetic parameters. For the crystallisation of $[Yb_2(BDC)_3(DMF)_2]\cdot H_2O$, we find one-dimensional crystal growth, entirely consistent with the needle-like crystals seen by microscopy, Figure S4.3a. The Bragg diffraction we observe shows little decrease in peak width beyond the first few patterns recorded, which would explain why we see no anisotropy in growth rate between different crystal directions. The value of *b* of ~ 10 min implies heterogeneous nucleation (predicted for b < 15),[2] while the probability of nucleation shows that nucleation occurs just in the earliest stage of reaction and does not extend far into the crystallisation period: this is consistent with the relatively large crystals seen by microscopy, which suggests that crystal growth has occurred at relatively few nucleation sites. An Arrhenius plot derived from the rate constants determined at the three temperatures studied, Figure 5.3b, allows activation energies for nucleation and for crystal growth to be determined. The results clearly show larger activation for crystal growth than for nucleation; such a situation has been found previously in MOF crystallisation, for example in the formation of the Co(II) form of MOF-74,[4] for NH_2-MIL-101(Al)[5] and for the formate modulated synthesis of ZIF-8.[6]

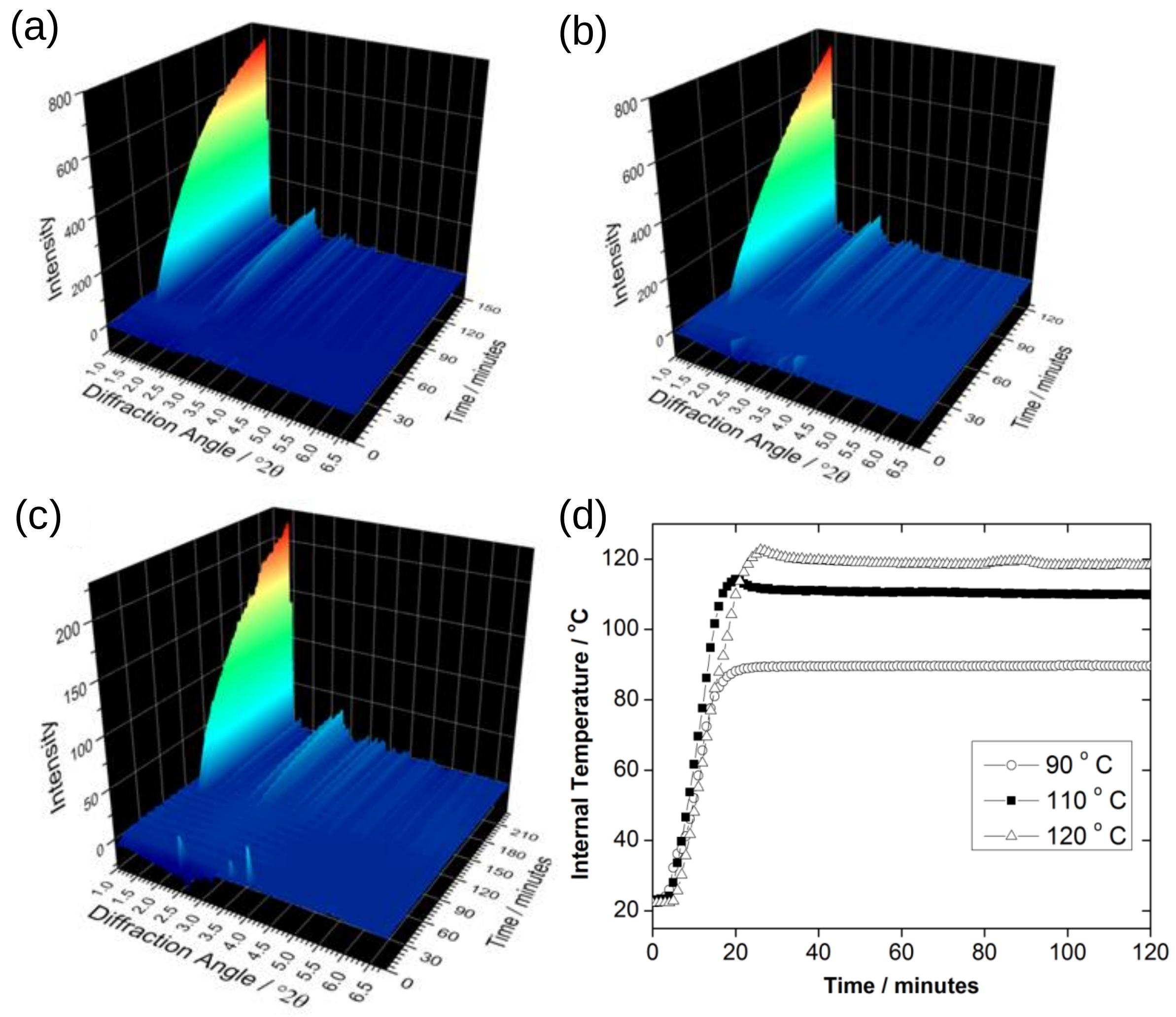

Figure S4.1: 3D contour maps (background subtracted patterns) of Bragg scattering measured during the crystallisation of [$Yb_2(BDC)_3(DMF)_2$]·H_2O at three different temperatures: (a) 120 °C, (b) 110 °C and (c) 90 °C. The internally measured temperature from each reaction is shown in (d).

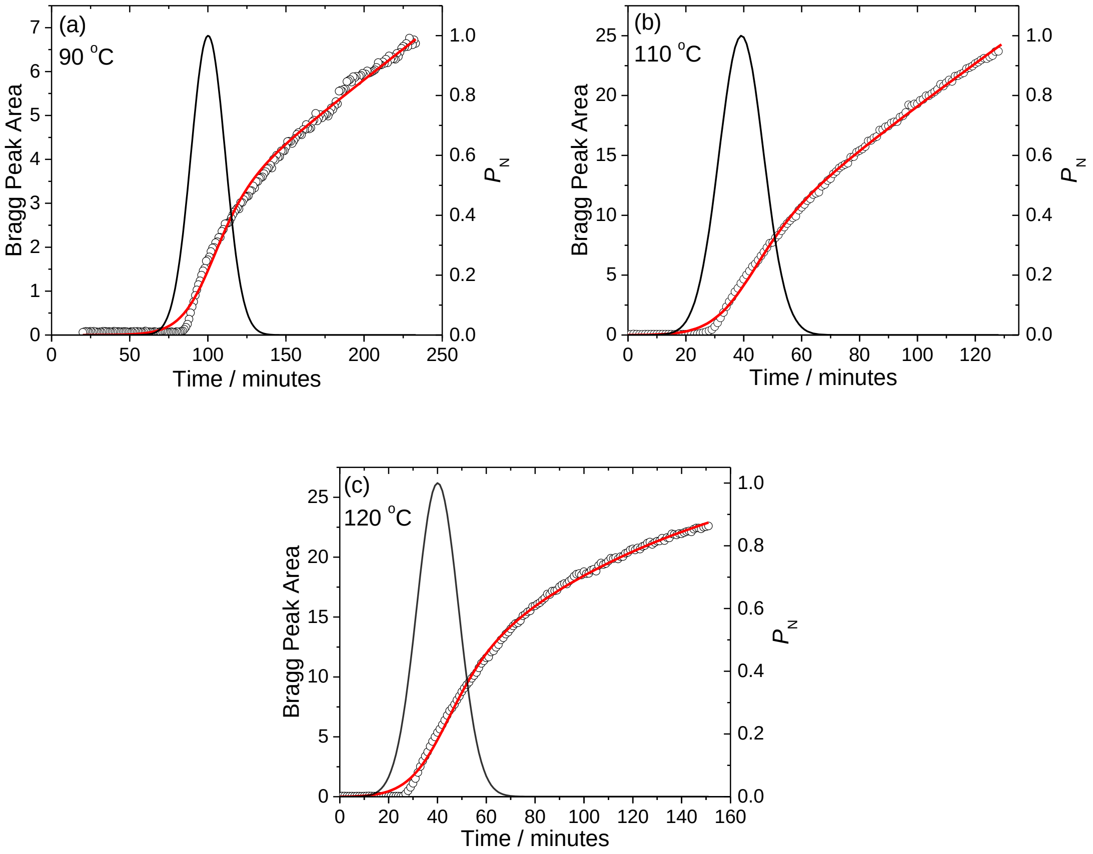

Figure S4.2: Bragg peak area curves (dotted lines) at three temperatures fitted by the Gualtieri kinetic model. The fitted Bragg peak area is shown by the red line and the probability of nucleation (*P_N*) shown by the black line.

Table S4.2: Kinetic parameters fitted to the crystallisation curves of $[Yb_2(BDC)_3(DMF)_2]\cdot H_2O$ using the Gualtieri nucleation-growth model

Temperature / °C	*a* / min	*b* / min	k_n / min^{-1}	k_g / min^{-1}
90	100.18 ± 0.48	10.95 ± 0.41	0.00998 ± 0.00005	0.00034 ± 0.00021
110	39.19 ± 0.32	7.70 ± 0.29	0.0255 ± 0.0002	0.00107 ± 0.00026
120	40.10 ± 0.30	8.53 ± 0.27	0.0249 ± 0.0002	0.00964 ± 0.00027

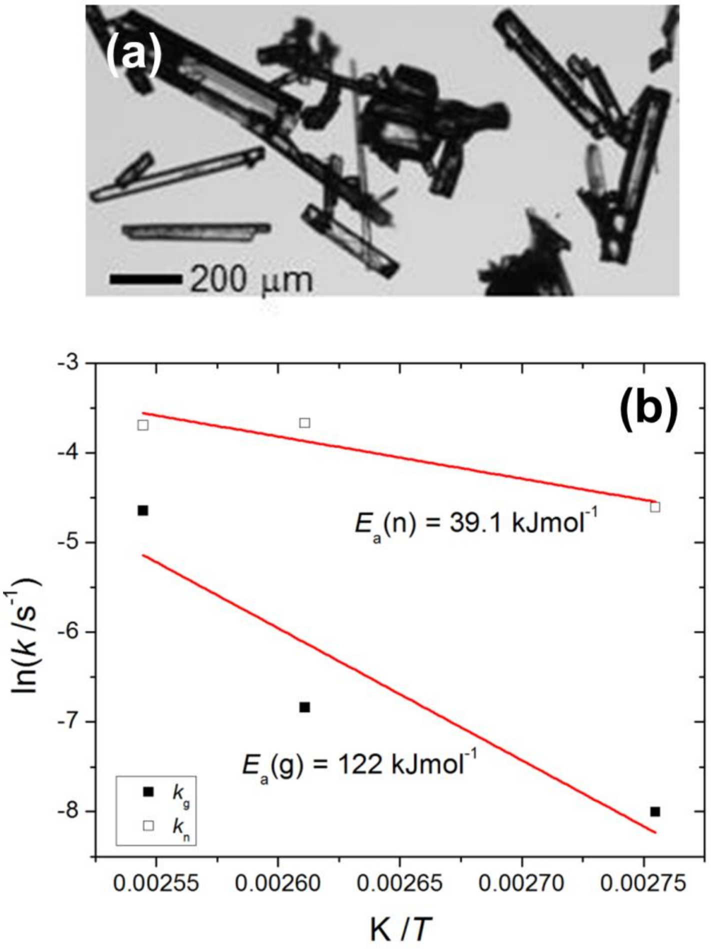

Figure S4.3: (a) Photograph of typical crystals of $[Yb_2(BDC)_3(DMF)_2]\cdot H_2O$ and (b) Arrhenius plots of rate constants for crystal growth (k_g) and nucleation (k_n).

S5: Study of the Desolvated Phase $Yb_2(BDC)_3$

Having studied the crystallisation of $[Yb_2(BDC)_3(DMF)_2]\cdot H_2O$, we investigated its stability towards calcination after synthesis, with the aim of removing occluded solvent to yield a porous structure. As noted above, thermogravimetric analysis shows sequential mass loss of water and DMF, with ultimate collapse to give Yb_2O_3 not occurring until above 500 °C. Thermodiffractometry is entirely consistent with the mass loss steps seen by TGA-DSC and at each step a material is formed that shows a distinctive powder XRD pattern, Figure S5.1. Although structure solution from these powder data was not possible, the material formed just above 300 °C undergoes a single phase change upon cooling with identical cooling profiles seen by thermodiffractometry under either air or dry nitrogen, suggesting that this material does not absorb water, Figure S5.2. This phase change is not a continuous one but occurs abruptly at around 150 °C upon cooling. We tentatively index the product as a mixture of phases, with a primary hexagonal phase with lattice parameters $a \approx 11.1$ Å, $c \approx 14.5$ Å, Figure S5.3. Elemental analysis (Medac Ltd, UK) of the material isolated at room temperature is consistent with the TGA and gives a chemical composition consistent with $Yb_2(BDC)_3$ (C: 33.92 %, 34.38 %(calc.); H: 1.68 %, 1.44 %(calc.)). Immersion in DMF did not regenerate the initial material, suggesting that this material no longer adopts the same connectivity, and the material is also hydrophobic with no evidence of water uptake after exposure to air as proven by TGA, which suggests the Yb in the material is coordinatively saturated, Figure S5.4. Nitrogen adsorption experiments with BET analysis were performed on samples of $[Yb_2(BDC)_3(DMF)_2]\cdot H_2O$ heated under vacuum at 100, 200 and 300 °C, Figure S6.1. The samples prepared at 200 and 300 °C display Type IV adsorption isotherms with H4-type hysteresis loops.[7] Low pressure hysteresis is indicative of strong interactions between the adsorbent and the adsorbate, possibly due to the presence of open metal sites. The samples activated at 200 and 300°C adsorb 80 and 100 cm^3 (STP) g^{-1} of nitrogen corresponding to BET surface areas of 218 and 305 m^2 g^{-1}, respectively. Thus we can conclude that the $Yb_2(BDC)_3$ produced is a microporous framework, having moderate surface area and with permanent porosity. Other $Ln_2(BDC)_3$ materials have been reported in the literature, although no crystal structures have been described and none of their powder patterns match the $Yb_2(BDC)_3$ we report here. Yaghi and co-workers reported that $Tb_2(BDC)_3(H_2O)_4$ could be dehydrated at elevated temperatures to form $Tb_2(BDC)_3$ with reabsorption of water upon cooling.[8] Daiguebonne *et al.* expanded on this and reported an isostructural series of $Ln_2(BDC)_3(H_2O)_4$ (Ln = La–Tm) materials that all displayed the same loss and reabsorption of water with temperature.[9] The materials containing the larger lanthanides (Ln = La–Eu) dehydrate with no overall change in their structure whereas those containing the smaller lanthanides (Ln = Tb–Tm) undergo a structural phase change. Pan *et al*. reported the synthesis of $[Er_2(BDC)_3(H_2O)_6]$. This material could be dehydrated to $Er_2(BDC)_3$ and but formed $[Er_2(BDC)_3(H_2O)_4]$ upon reabsorption of water.[10]

Thermodiffraction experiments were carried out using a Bruker D8 powder diffraction operating with Cu Kα1/2 radiation and fitted with an HTK900 gas chamber and VÅNTEC-1 detector. Patterns were recorded in

static air on heating from room temperature to 600 °C in intervals of 20 °C with a 10 minute equilibration time before scans lasting 10 minutes were made.

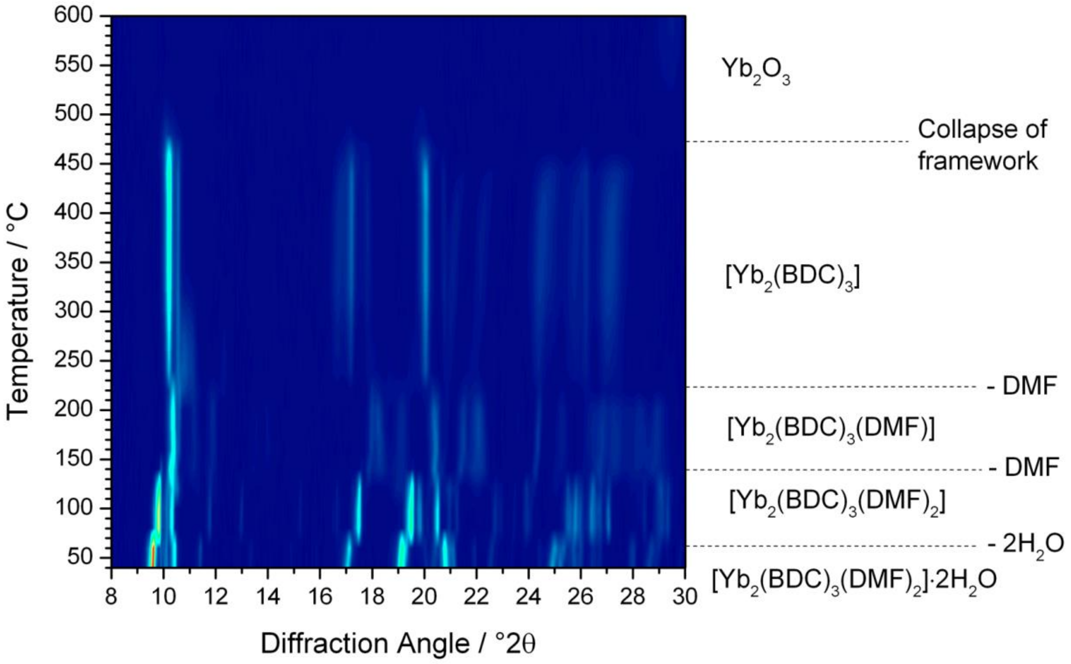

Figure S5.1: Thermodiffraction measure during heating of $[Yb_2(BDC)_3(DMF)_2]\cdot H_2O$, with phases assigned based on thermogravimetric analysis in Figure S3.1.

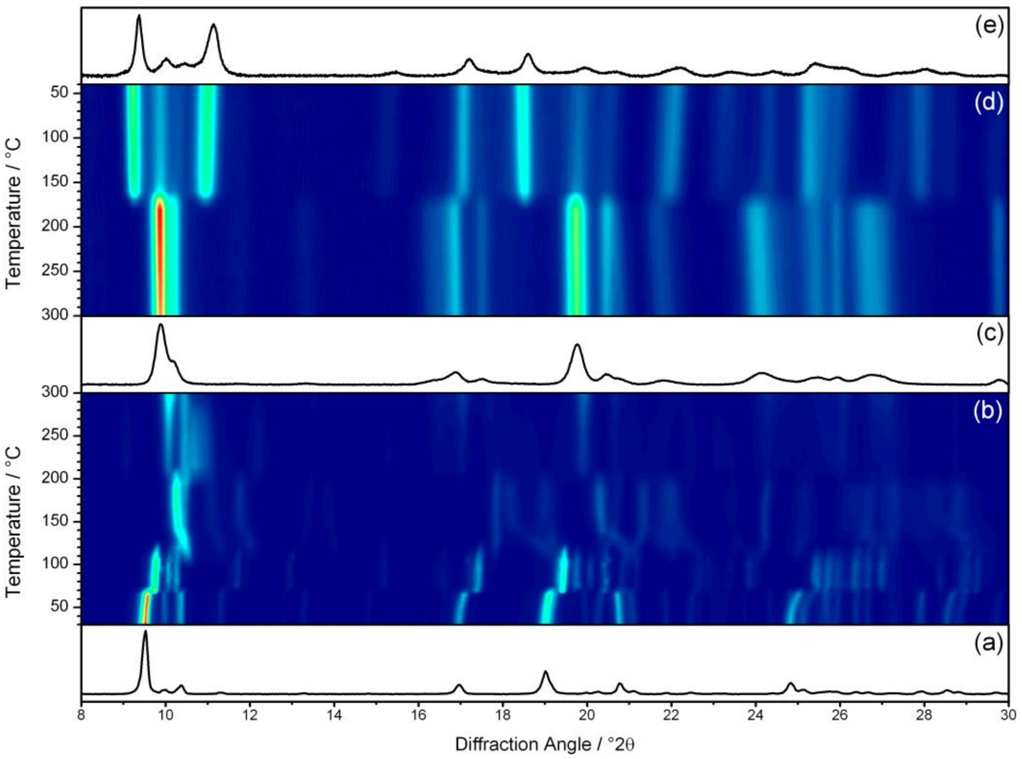

Figure S5.2: (a) PXRD pattern of [$Yb_2(BDC)_3(DMF)_2$]·H_2O at 30 °C; (b) thermodiffractometry of [$Yb_2(BDC)_3(DMF)_2$]·H_2O heating to 300 °C in air; (c) PXRD pattern of at 300 °C; (d) thermodiffractometry of cooling back to 30 °C in air; (e) PXRD pattern of after cooling

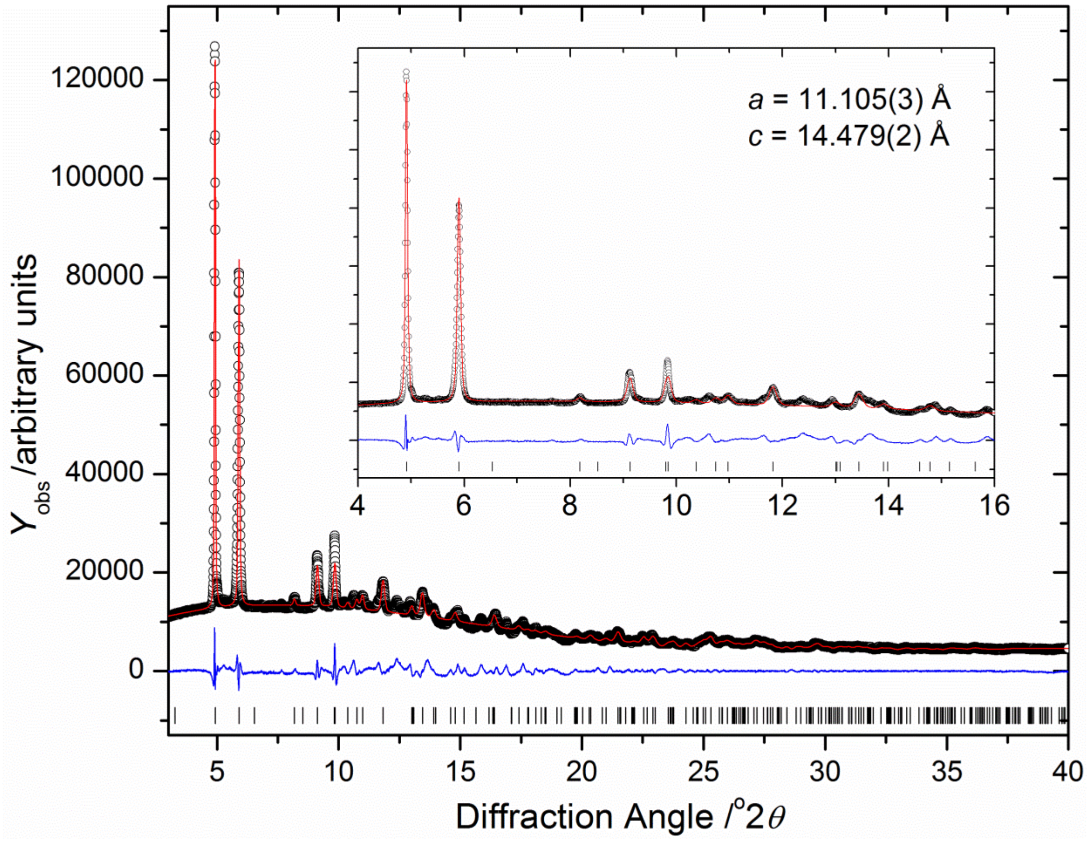

Figure S5.3: Pawley fit to PXRD pattern of $Yb_2(BDC)_3$ indexed with a hexagonal unit cell. Data were collected on Beamline I11 at Diamond (λ = 0.825174 Å). (Data are points, red line the fit and the blue line the difference curve.) The sample was produced from $[Yb_2(BDC)_3(DMF)_2]\cdot H_2O$ using a heating and cooling rate of 0.2 $°C\cdot s^{-1}$ and a dwell time of 6 hours at 300 °C.

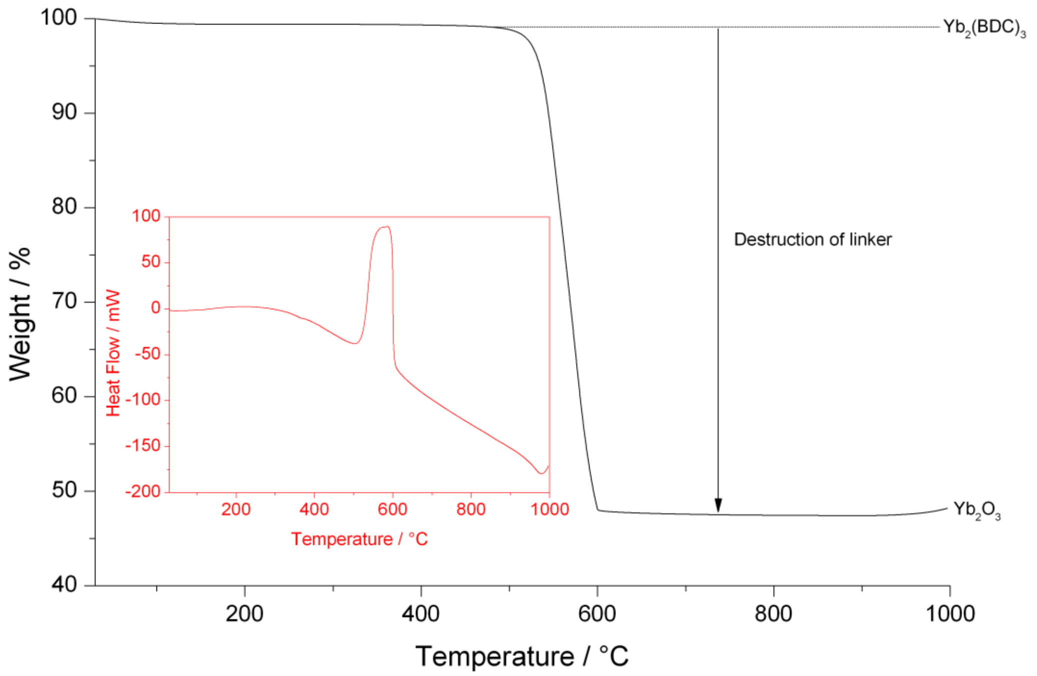

Figure S5.4: Main: TGA of $Yb_2(BDC)_3$ in air; Inset: DSC curve of $Yb_2(BDC)_3$ over same temperature range

S6: BET Analysis of nitrogen adsorption from $Yb_2(BDC)_3$

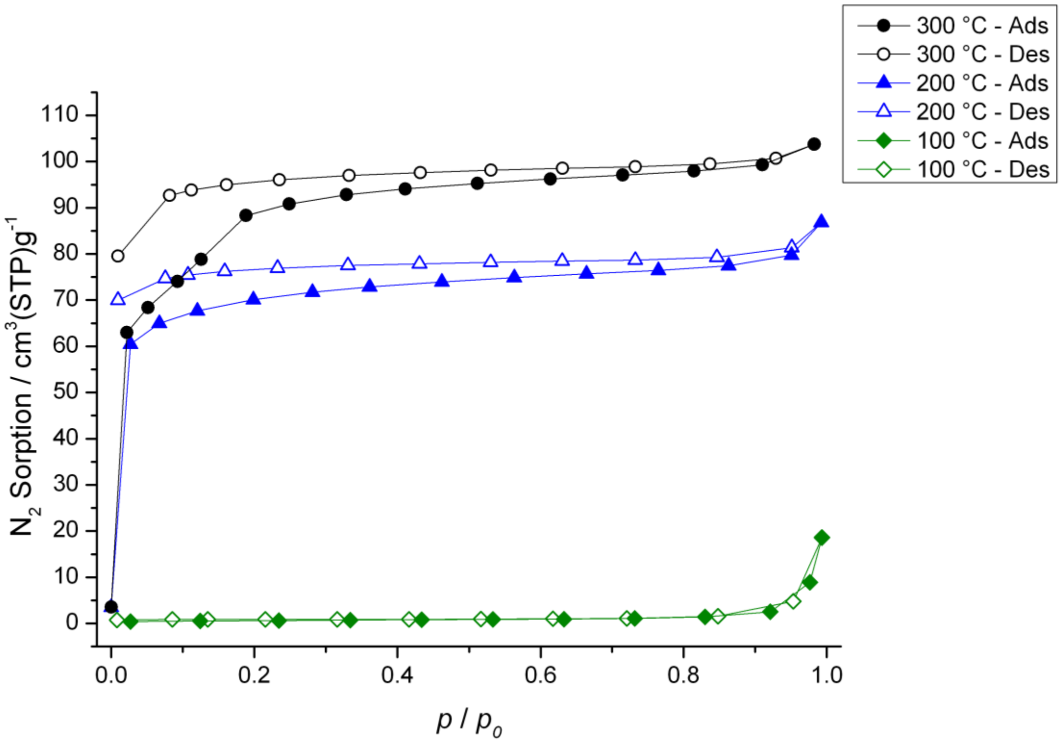

Figure S6.1: N_2 sorption isotherms of $[Yb_2(BDC)_3(DMF)_2]\cdot H_2O$ after heating under vacuum at various temperatures. Filled symbols: adsorption; Empty symbols: desorption.

S7: *In Situ* Powder XRD – Rietveld analysis

Rietveld fits were attempted using the structural model obtained from SXCRD (Figure S7.1). The model did not prove robust enough to allow full structural refinement, but captures the main features of the PXRD data. Background and peak shape parameters were obtained from the previous Pawley refinement. In this case, peaks from the PEEK sample container were modelled based on a known orthorhombic unit cell.[11]

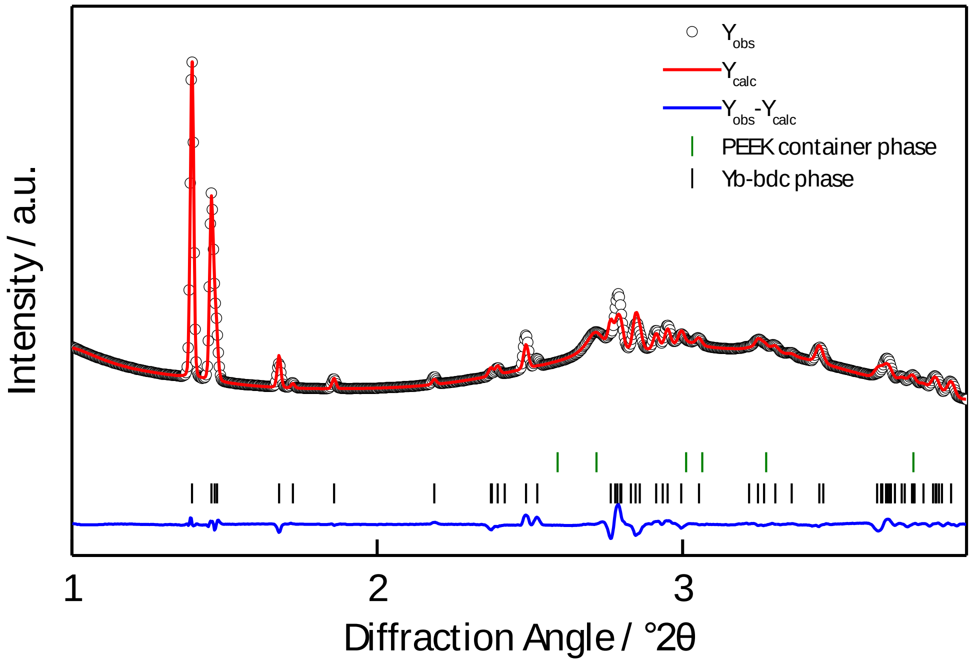

Figure S7.1: Example of Rietveld fit of *in situ* data from 120 °C reaction at 150 min

Sequential Rietveld fits were run on the *in situ* data with most structural parameters fixed. The background, peak shape and lattice parameters were allowed to freely refine. The occupancy of all atoms in the DMF moiety was linked to a single parameter and allowed to freely refine, except for the oxygen atom which was fixed at 1, as this site is occupied by oxygen regardless of H_2O or DMF coordination. The PEEK was modelled as a Pawley phase with peak shape fixed, cell parameters constrained within 1 % and intensity freely refining. Refinement details are provided in Table S7.1.

In Figure S7.2 below, the change in DMF occupancy parameter is compared to the ratio of (200) and (110) peak areas obtained from Pawley refinement, which show a very similar trend. This is consistent with the fact that the DMF electron density lies primarily on the crystallographic (200) plane.

Table S7.1: Details of Rietveld refinement of *in situ* data from 120 °C reaction at 150 min

**Parameter**	**Value**
Crystal system	Monoclinic
a / Å	18.546(2)
b / Å	10.8154(5)
c / Å	18.051(3)
β / °	108.00(1)
V / $Å^3$	3492.9(6)
Space group	*C* 1 2/*c* 1
Laue group	2/*mb*
λ / Å	0.2242
Zero point	Not refined (image plate data)
Number of variables	71
R_p / %	1.14
wR_p / %	2.17
χ^2	0.12

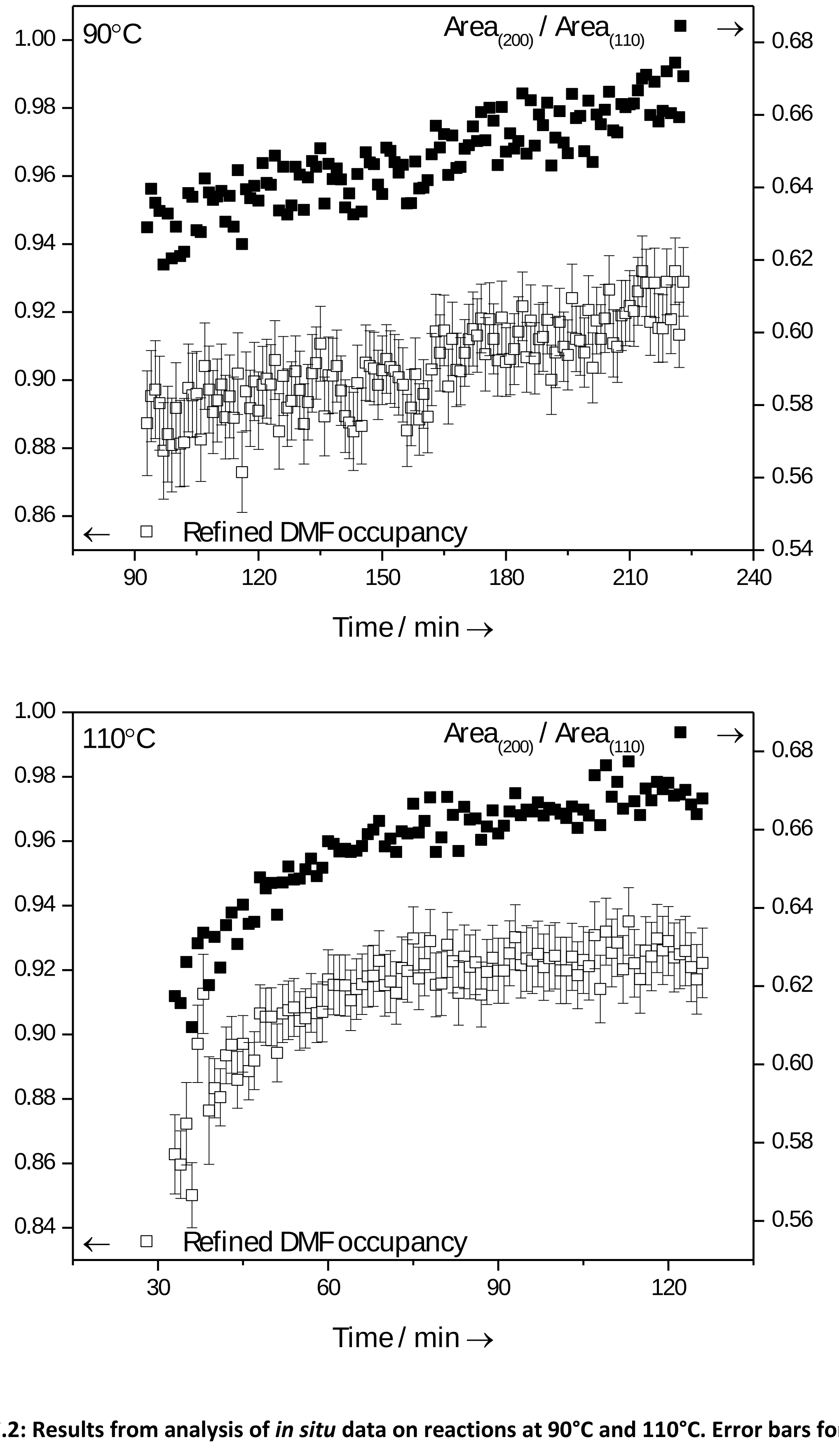

Figure S7.2: Results from analysis of *in situ* data on reactions at 90°C and 110°C. Error bars for Pawley analysis are smaller that data points.

S8: References

[1] A. Coelho, *TOPAS-Academic V5, Coelho Software* **2012**.

[2] *CRC Handbook of Basic Tables for Chemical Analysis*, Third Ed (2010) CRC Press p 590

[3] A.F. Gualtieri, *Phys. Chem. Miner.* **2001**, *28*, 719.

[4] R. El Osta, M. Feyand, N. Stock, F. Millange, R.I. Walton, *Powder Diffr.* **2013**, *28*, S256.

[5] E. Stavitski, M. Goesten, J. Juan-Alcaniz, A. Martinez-Joaristi, P. Serra-Crespo, A.V. Petukhov, J. Gascon, F. Kapteijn, *Angew. Chem., Int. Ed.* **2011**, *50*, 9624.

[6] J. Cravillon, C.A. Schroder, H. Bux, A. Rothkirch, J. Caro, M. Wiebcke, *CrystEngComm* **2012**, *14*, 492.

[7] K.S.W. Sing, D.H. Everett, R.A.W. Haul, L. Moscou, R.A. Pierotti, J. Rouquerol, T. Siemieniewska, *Pure Appl. Chem.* **1985**, *57*, 603.

[8] T.M. Reineke, M. Eddaoudi, M. Fehr, D. Kelley, O.M. Yaghi, *J. Am. Chem. Soc.* **1999**, *121*, 1651.

[9] C. Daiguebonne, N. Kerbellec, K. Bernot, Y. Gerault, A. Deluzet, O. Guillou, *Inorg. Chem.* **2006**, *45*, 5399.

[10] L. Pan, N.W. Zheng, Y.G. Wu, S. Nan, R.Y. Yang, X.Y. Huang, J. Li, *Inorg. Chem.* **2001**, *40*, 828.

[11] A. V. Fratini, E. M. Cross, R. B. Whitaker, W. W. Adams, *Polymer* **1986**, *27*, 861–865.
